# The Formation and Invariance of Canine Nose Pattern of Beagle Dogs from Early Puppy to Young Adult Periods

**DOI:** 10.3390/ani11092664

**Published:** 2021-09-10

**Authors:** Hyeong In Choi, Yoonsuk Lee, Hyunjin Shin, Sungjin Lee, Stephanie Sujin Choi, Chang Yong Han, Song-Hwa Kwon

**Affiliations:** 1 Department of Mathematics, Seoul National University, Seoul 08826, Korea; hichoi@snu.ac.kr; 2 iSciLab Corporation, Seoul 08791, Korea; choistephanies@iscilab.com; 3 College of Veterinary Medicine, Konkuk University, Seoul 05029, Korea; leeyoons@konkuk.ac.kr (Y.L.); shj1051@konkuk.ac.kr (H.S.); 4 Department of Mathematics, Daejin University, Pocheon 11159, Korea; hyper@daejin.ac.kr; 5 Department of Applied Mathematics, Kyung Hee University, Yongin 17104, Korea; cyhan@khu.ac.kr; 6 Department of Mathematics, The Catholic University of Korea, Bucheon 14662, Korea

**Keywords:** canine, dog, nose, nose pattern, nose print, invariance of nose pattern, biometrics, template, Gabor transform, Hamming distance

## Abstract

**Simple Summary:**

In this paper, we examine whether the canine nose pattern, which is an interlocking pattern of beads and grooves on a dog’s nose, is unique to each individual animal. For this purpose, the nose images of ten beagle dogs were taken every month for the ten-month period starting from month two and ending in month eleven. Six of them are siblings born of one dam and the other four of another dam. In this longitudinal study, the canine nose patterns of these ten dogs are examined visually and by a biometric algorithm to determine whether the canine nose patterns in two images of the same dog taken at different time remain the same and whether two images of different dogs are indeed different regardless of when the images are taken. It is found that the canine nose pattern of the beagle dog is fully formed at the second month after birth, that this nose pattern stays invariant, and that the canine nose pattern is indeed unique to each animal. Our finding confirms and enhances the claims of earlier works that the canine nose pattern is indeed unique to each animal, and could be used as a unique biometric marker.

**Abstract:**

The formation and invariance of the canine nose pattern is studied. Nose images of ten beagle dogs were collected for ten months from month two to month eleven. The nose patterns in these images are examined visually and by a biometric algorithm. It is found that the canine nose pattern is fully formed at the end of the second month since birth and remains invariant until the end of the eleventh month. This study also strongly indicates that the canine nose pattern can be used as a unique biometric marker for each individual dog.

## 1. Introduction

Dogs are now accepted as part of families. For many people, the affectionate bond between humans and animals is strong and enduring. Since many dog lovers believe such a bond develops faster and stronger if a puppy becomes accustomed to its owner at as early an age as possible, they want to adopt dogs from breeders as soon as they are ready. The earliest nrmally accepted adoption time for puppies is about three months after birth, although some people want to push this to around two months. Adoption before that time is normally not recommended, because it is better for a puppy to be nurtured for eight to twelve weeks in an environment with its dam and siblings. It takes about six weeks for a puppy to be weaned from its dam’s milk, and it will grow to be a healthy and happy animal in a secure nurturing environment with its dam and the litter of siblings. Vaccination is also an important requirement for adoption. Breeders usually give puppies their first vaccination between six and eight weeks after birth, and give the second between two and four weeks afterwards. There are several other vaccinations to be administered during a dog’s lifetime. The current normal practice is to put up puppies for adoption starting at three months after birth, although there is not insignificant market demand to allow this after two months. Before their release to new owners, dogs undergo a certain registration process. The most common method of registration is microchip insertion into the body of a dog. There are, however, many public inquiries looking for alternatives to microchip insertion. Since human biometric technologies such as fingerprint, face, iris and vein recognition are now widely used, many in the public as well as in governments are quite interested in knowing if such biometric technologies are applicable to dogs.

The objective of this paper is to focus on the canine nose pattern (nose print) by studying if it can be used similarly to the human fingerprint as a unique biometric marker for each individual dog. If so, it will open up many possibilities in animal welfare. For example, a dog’s nose pattern image can be captured by anyone with a smart phone camera. This image can then be used for animal identity registration, and the animal’s identity, once registered, could be retrieved or verified very easily with a smart phone. Animal registration can be easily done this way, even in a developing country with no microchip insertion facility nearby, thereby solving the common problem of low registration rates with minimal cost. Even with a widespread use of subcutaneous microchips, this non-invasive method using only nose pattern images can serve as a back-up that co-exists with the microchip standard. Microchips are also known to have some side effects to animals in certain cases, as observed by various studies. Cancers in dogs caused by microchip insertion are reported in [1,2], and granulomatous inflammatory response was also reported in [3]. Similar types of cancers are also reported for cats that are inserted with microchips [4,5]. Cancers caused by microchip insertion in rodents are also reported in [6,7,8,9]. Various spinal cord injuries after microchip insertion are also reported, as in [10,11], and, in some cases, tetraparesis may occur [12]. The case of a microchip’s migration to the brainstem is also reported [13]. For more cases, one may consult the paper by Swift that keeps track of adverse microchip reactions [14]. It should be noted that such reported side effects due to microchip insertion are rather rare. Nonetheless, this acts as a psychological hindrance to microchip insertion for some people. For these people, this non-invasive method may induce them to overcome such fears. In this sense, canine nose biometrics may have a place as a complementary or back-up means of microchip insertion.

To reach this goal, this paper focuses on the following two fundamental questions: (1) if the canine nose pattern is properly formed at the earliest proposed adoption time, namely, at two months after birth; (2) if this nose pattern remains invariant throughout a puppy’s growth to a young adult dog. The findings of this paper give affirmative answers to both of these questions.

The time period with which this paper is concerned is the ten month period beginning at the end of the second month of a dog’s birth (the second mensiversary) and ending at the end of the eleventh month since birth (the eleventh mensiversary). The choice of the second mensiversary is dictated by the market reality of possible early adoption, as explained above; the eleventh mensiversary is chosen because it is around that time a puppy enters into the adult stage of life, from which point the stability of biological traits largely prevails for the remainder of its life.

The animal nose print has been studied for a long time. Research goes as far back as the early 1920s. For instance, in 1922, W. Petersen studied the nose prints of more than 350 cows and concluded that no two animals have an identical pattern and that nose prints afford a positive means of identification [15], and Baranov et al. [16] dealt with the estimation of genetic parameters of muzzle patterns in cattle and with breed differences. There is a sizable body of literature on the bovine (cattle) nose print.

However, the literature on the canine nose print is rather scant. Coldea [17] made a comparative microscopic study on both the human finger skin and the canine nose and reported that both contained prints specific to each individual. Miller’s Anatomy of the Dog, 4th Edition, by Evans and de Lahunta, citing Horning et al. [18], states that “nose prints, similar to finger prints, can be used to distinguish between individuals” [19], p. 64. Another textbook by Budras et al. states that “The dermis forms distinct papillae. The epidermis is strikingly thin, and its superficial, cornified layer (stratum corneum) consists of hard ’horn’ (hard cornified epidermis) that exhibits a polygonal pattern. The surface pattern is individually specific and for this reason serves to identify the individual animal” [20], p. 6. There are several algorithm papers for cattle nose print identification that may be relevant in our context. Earlier work was conducted by Barry et al. [21], who used the muzzle pattern as a means of cattle identification, and Noviyanto and Arymurthy [22,23], have developed an automatic cattle identification method using muzzle photos. Subsequently, many related papers by various authors have appeared. See: [24,25,26,27,28].

However, all of these studies and the claims therein are based on studies on samples taken in a short period of time, and there were no follow-up studies for a longer subsequent timespan. In this sense, they can be dubbed cross-sectional studies. In comparison, the objectives of this paper dictate that each individual animal has to be followed up for a longer period for possible changes or variations; in other words, our work has to be a longitudinal study for a sufficiently longer period of time. As far as we are aware, this kind longitudinal study on the formation and invariance of the canine nose pattern is the first of its kind. We believe that this is one of the main contributions of our work.

Another point worth mentioning is this paper’s focus on the siblings. As pointed out in the References [17,18,19,20], the nose pattern of dogs is known to be unique to each individual animal. However, there has been no detailed study on whether canine siblings actually do have different nose patterns. If there is any chance that the canine nose pattern is not unique, logic would suggest that evidence of such is more likely to manifest among siblings that have a high degree of genetic similarity. Therefore, since checking for pattern uniqueness among siblings presents a stronger test case, we have deliberately chosen to focus on the sibling groups of two dams of the same breed, i.e., the beagle dogs. Furthermore, the inclusion of two dams helps discount the potential of anomaly in a single family as a factor. The findings of this paper are that every individual has a different nose pattern. This confirms and enhances the claims of earlier works by others, as in [17,18,19,20], that the canine nose pattern is indeed unique to each animal and may be used as a unique biometric marker.

## 2. Canine Nose Pattern and its Biological Basis

**Figure 1:**
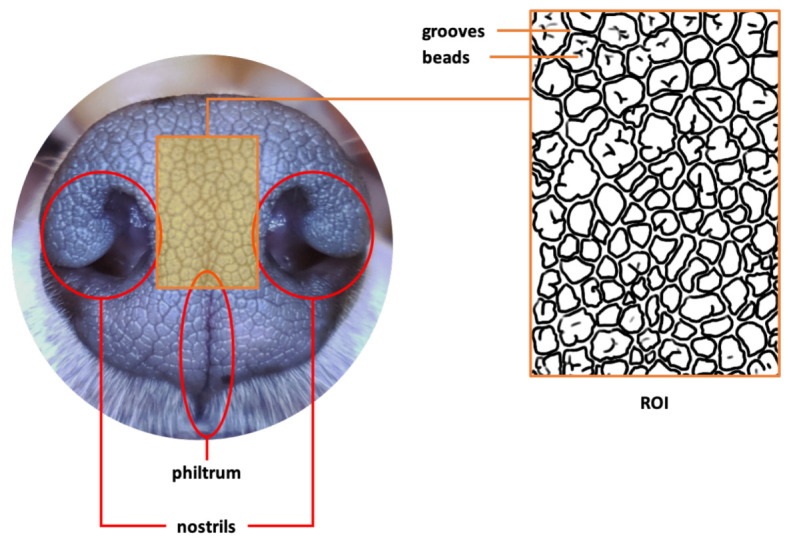
Canine Nose and Nose Pattern in ROI (Copyright 2021 by the authors).

Figure 1 shows an example of a typical image of a canine nose pattern. The boxed middle area, called the Region of Interest (ROI), is enlarged and illustrated as an outline sketch. As can be seen here, the canine nose has a complex interlocking pattern of obtruded regions, called *beads*, and the sunken narrow ribbon-like regions between the beads, called *grooves*. This complex interlocking pattern of beads and grooves is a biometric marker that is unique to each individual animal.

The canine nose pattern is comparable to the human fingerprint in its biological origin. First, human fingerprints are essentially collections of connected friction ridges, which are raised portions of the epidermis on the digits, of ectodermal embryological origin. These epidermal friction ridges are the result of the interface between the cornified layer of the epidermis and the pattern of the underlying dermal papillae that project upwards into the epidermis. Although no exact mechanism of ridge formation has been proven to date, the general theory hypothesizes a connection between the basal layer of the epidermis and the dermis during early embryonic development. The basal layer of the epidermis, which is adjacent to the dermis, consists of columnar cells perpendicular to the skin surface. When the basal layer becomes undulated during the tenth to thirteenth week in embryonic development, so do the columnar cells, indenting folds into the dermis. These folds become the primary ridges and give way to the eventual irregular line of attachment between the dermis and epidermis at the basis of friction ridges throughout [29].

Similarly, the distinct nose patterns on certain mammals are of ectodermal embryological origin and determined by genetic and environmental factors in utero. The nasal plane (planum nasale) exhibits plaque-like patterns which, like the friction ridges on fingertips, are caused by the underlying interface between the dermal papillae and the interpapillary pegs of the epidermis. For example, the dermis of the canine planum nasale consists of a deeper reticular layer and a papillary layer, upon which rests the thin epidermis with a thin cornified layer (stratum corneum) at the most exterior, which presents a polygonal pattern that is characteristic of each individual [20]. While there are certain structural disparities in the nasal epidermis between different species—for instance, the bovine nose is glandular while the canine is not—the interdigitation of dermal papillae with epidermal projections is a consistent feature in those with visible surface patterning of the nose [30].

## 3. Materials and Methods

As explained in the Introduction, the two goals of this study are to examine: (1) if the canine nose pattern is fully formed at the second mensiversary; (2) if this nose pattern stays invariant throughout a dog’s life and could be used as a biometric marker. To achieve this goal, we have collected data by following the procedure below. This study was conducted at Konkuk University according to the guidelines of the Institutional Animal Care and Use Committee, and was approved by Konkuk University’s Animal Experiment Ethics Committee under the approval number KU 19203.

- Birth of puppies
  On 6 Novemebr 2019, the staff of Konkuk University Veterinary Medical Teaching Hospital procured two pregnant female beagle dogs that gave birth to ten puppies on 19 November 2019. Six of the ten puppies were born of one dam and four of the other. Genetic identification of individual puppies was performed with the canine genotype panel 2.1 kit (ThermoFisher) using buccal swabs.
- Capturing nose images
  After taking care of necessary precautions such as vaccinations, the staff started to capture nose images of each of the ten puppies on 16 January 2019 (the second mensiversary). Afterwards, nose images of the ten puppies were taken every month for the ten-month period ending on 21 October 2020 (the eleventh mensiversary). The exact dates are recorded in Table 1.

The choice of the second mensiversary as the start date was to examine Goal (1). This experiment ends at the eleventh mensiversary, because it is around that time a puppy enters into the adult stage of its life, from which point the stability of biological traits largely prevails for the remainder of its life, hence achieving Goal (2).

**Figure A1:**
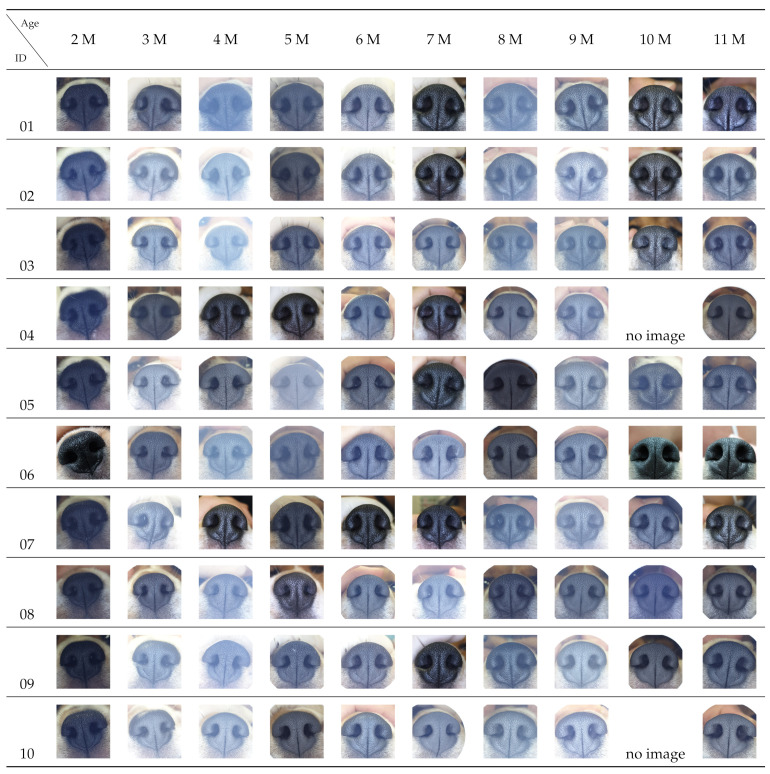
All images.

The nose images of each puppy taken at each mensiversary are shown in Figure A1 in Appendix A. Two images are missing because the new owners who adopted the two puppies around the third mensiversary could not bring them in for image-taking on the appointed day.

## 4. Results

The formation of the nose pattern at the second mensiversary and its invariance in the subsequent months can be checked in two ways: by direct visual examination and by algorithmic verification.

### 4.1. Visual Examination

Figure A2, Figure A3, Figure A4, Figure A5, Figure A6, Figure A7, Figure A8, Figure A9, Figure A10 and Figure A11 show the ROIs of the ten dogs taken during the ten-month period. As written in the caption of each image, the dog in Figure A2 has ID 01, and that in Figure A3 has ID 02, and so on.

**Figure A2:**
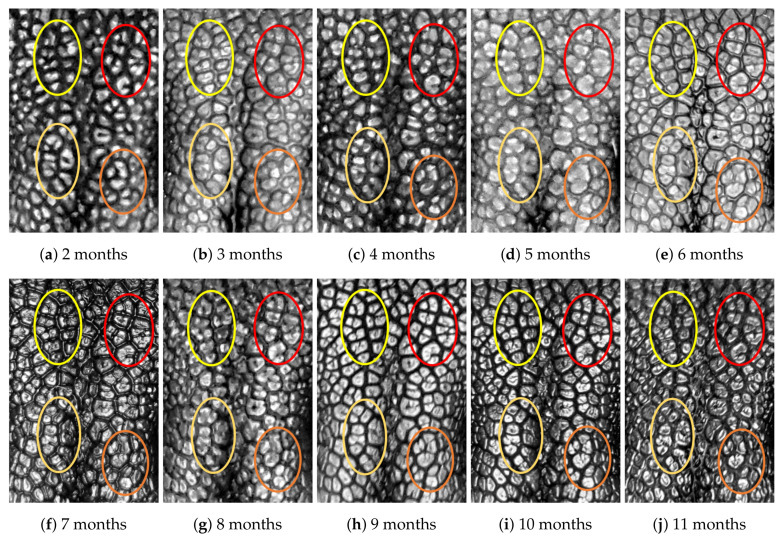
Nose images from 2 months to 11 months of age for the same subject (ID 01): beads and grooves in ovals with the same color have the same patterns.

**Figure A3:**
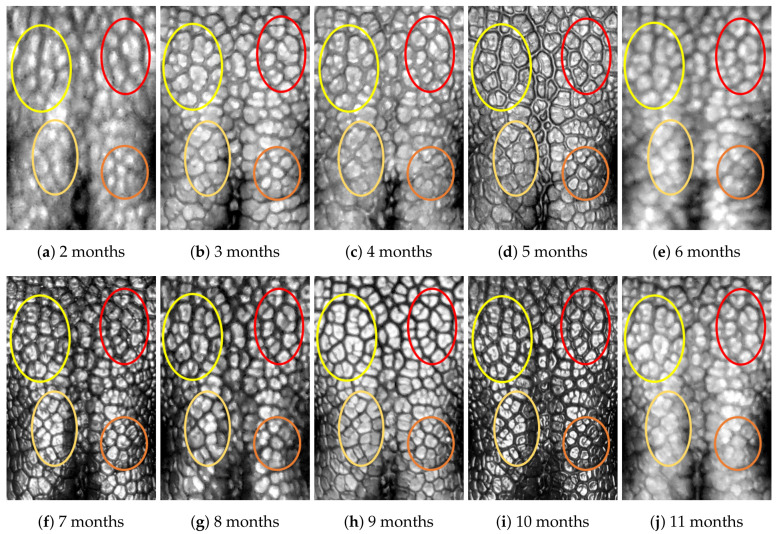
Nose images from 2 months to 11 months of age for the same subject (ID 02): beads and grooves in ovals with the same color have the same patterns.

**Figure A4:**
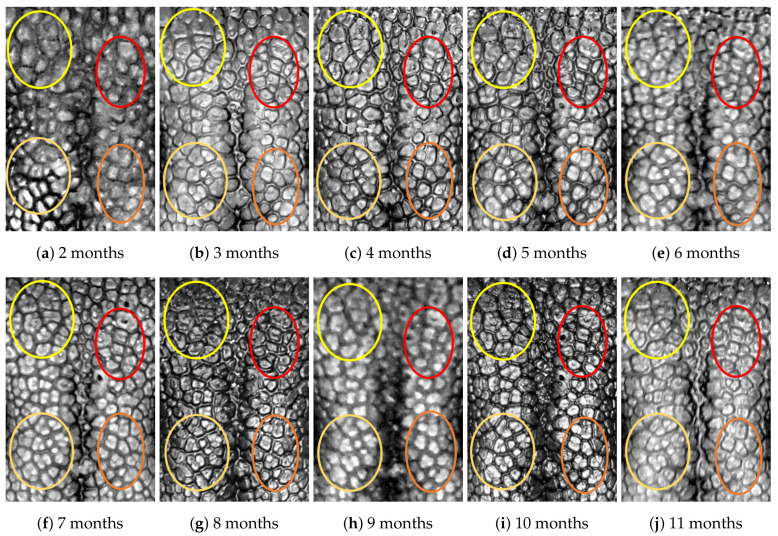
Nose images from 2 months to 11 months of age for the same subject (ID 03): beads and grooves in ovals with the same color have the same patterns.

**Figure A5:**
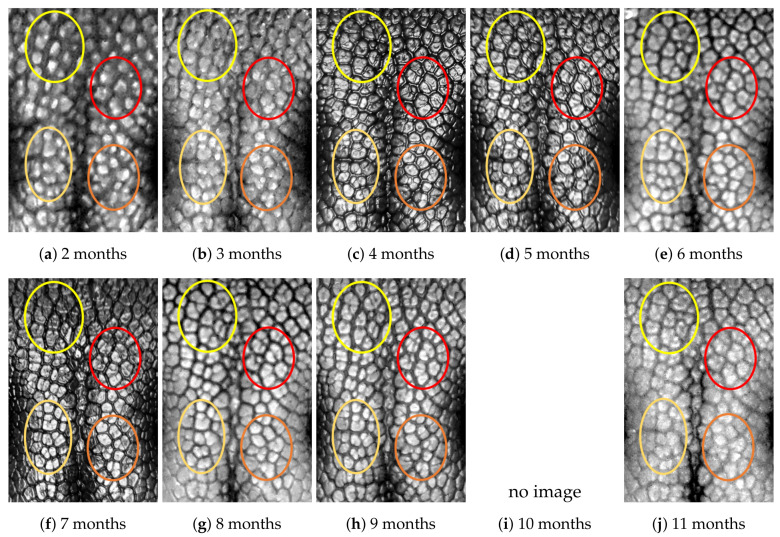
Nose images from 2 months to 11 months of age for the same subject (ID 04): beads and grooves in ovals with the same color have the same patterns.

**Figure A6:**
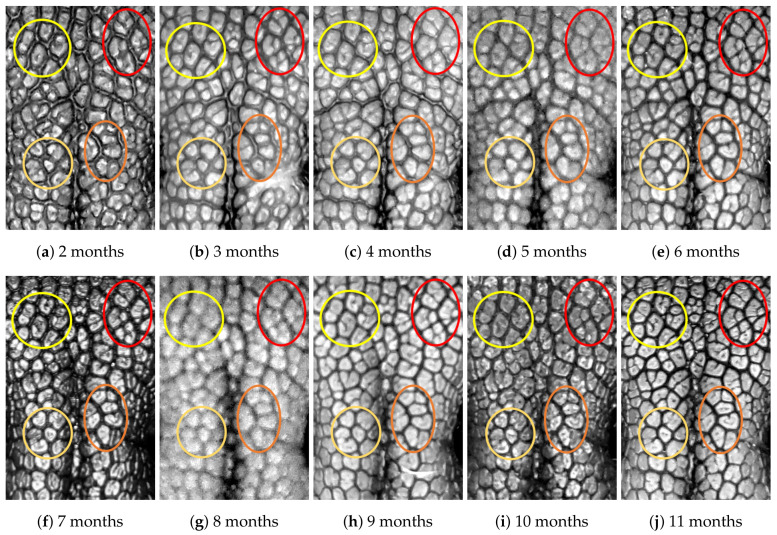
Nose images from 2 months to 11 months of age for the same subject (ID 05): beads and grooves in ovals with the same color have the same patterns.

**Figure A7:**
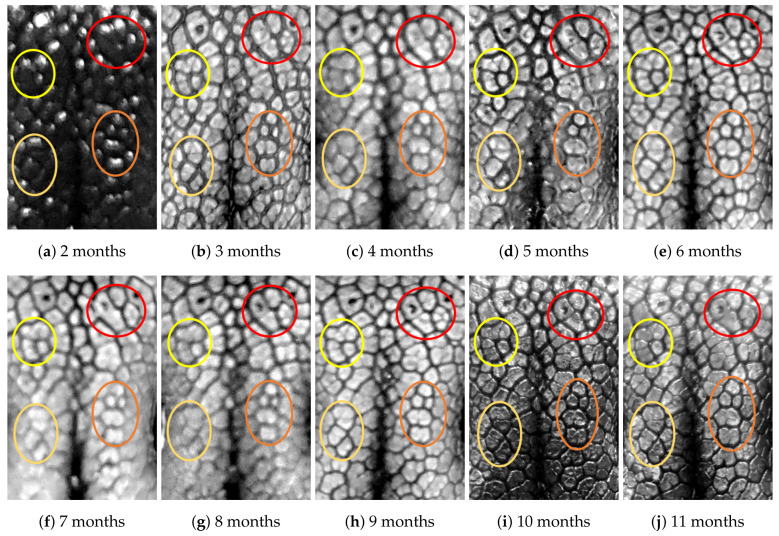
Nose images from 2 months to 11 months of age for a same subject (ID 06): beads and grooves in ovals with the same color have the same patterns.

**Figure A8:**
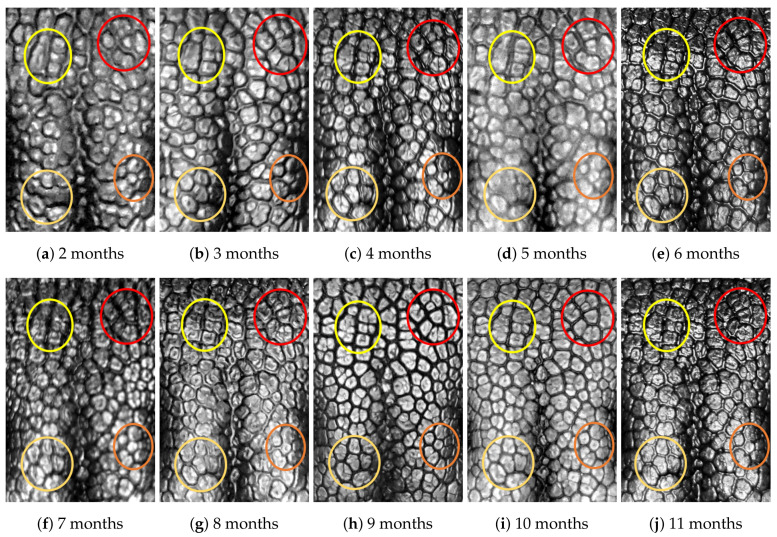
Nose images from 2 months to 11 months of age for the same subject (ID 07): beads and grooves in ovals with the same color have the same patterns.

**Figure A9:**
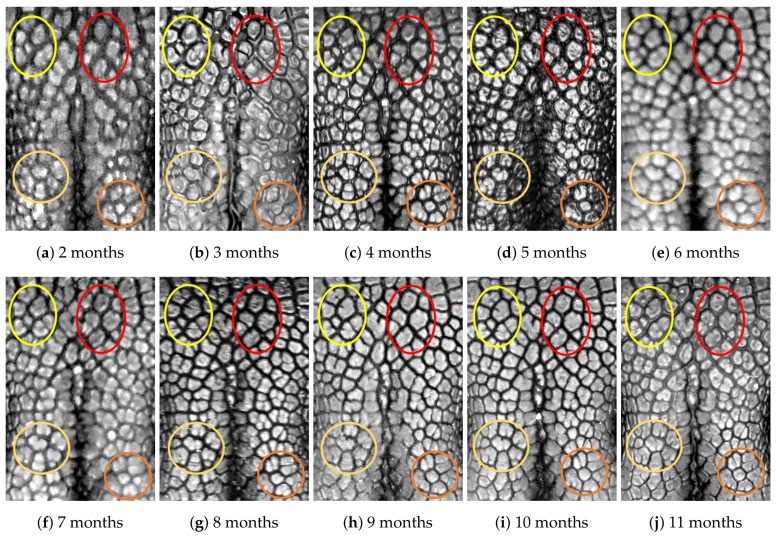
Nose images from 2 months to 11 months of age for the same subject (ID 08): beads and grooves in ovals with the same color have the same patterns.

**Figure A10:**
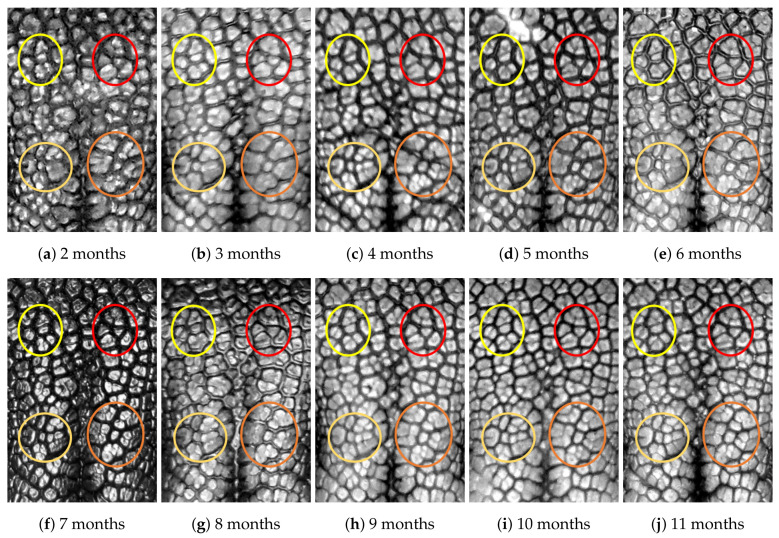
Nose images from 2 months to 11 months of age for the same subject (ID 09): beads and grooves in ovals with the same color have the same patterns.

**Figure A11:**
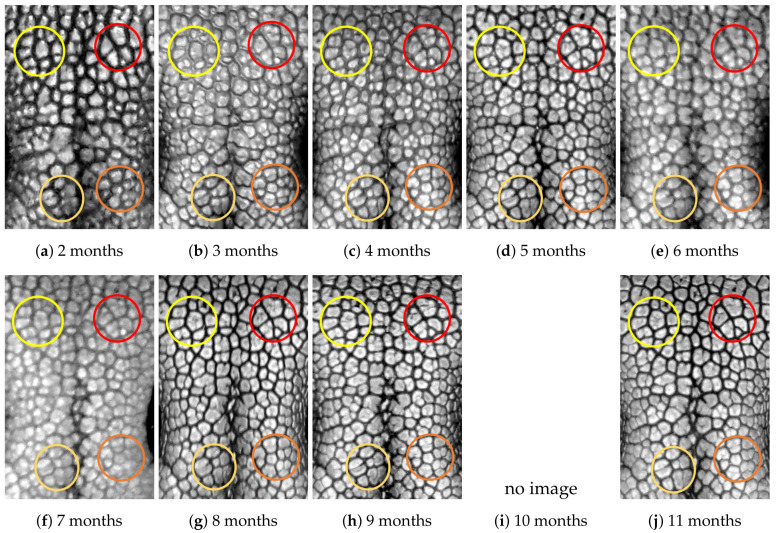
Nose images from 2 months to 11 months of age for the same subject (ID 10): beads and grooves in ovals with the same color have the same patterns.

First, let us visually check if a dog’s nose pattern is fully formed at the second mensiversary and if this nose pattern remains the same during the ten-month observation period. At a first glance, it is not easy for untrained eyes to discern the intricacies of the interlocking pattern of the canine nose pattern to determine if two canine nose patterns look the same or not. Therefore, as a visual aid, we have overlaid four ovals on each of the ROI images in Figure A2, Figure A3, Figure A4, Figure A5, Figure A6, Figure A7, Figure A8, Figure A9, Figure A10 and Figure A11. These ovals are useful visual aids to help illustrate the similarities and differences between the images. However, it should be emphasized that the ovals are in no way necessary for pattern recognition. Specifically, they are not landmarks nor are they something like the minutiae in human fingerprint image analysis. It should also be noted that once one gets used to canine nose patterns, one can do without any such oval aids. With these comments on ovals understood, let us now proceed to the visual analysis of canine nose patterns.

Upon examining the images in Figure A2, it can clearly be seen that the pattern in the top left yellow oval area remains more or less the same when compared with that in each of the other images of the ten-month period. Similarly, the patterns in each of the other three ovals can be seen to be more or less the same. One can observe similar invariance in the nose pattern of each of the ten dogs. From these observations, one can conclude that a dog’s nose pattern is fully formed at the second mensiversary and that this nose pattern remains the same during the ten-month observation period.

Next, let us compare the nose patterns of different dogs. For example, Figure A2 and Figure A3 show the nose patterns of two different dogs with IDs 01 and 02. Note that the relative locations of the four ovals in the two sets of images are different, and the patterns in the regions within the ovals are also different. Upon closer examination of any pair of images of two different dogs, one can easily see that similar differences persist in all pairs of nose images coming from different dogs. This indicates that the nose pattern can be used as a unique biometric marker for each individual dog.

### 4.2. Algorithmic Verification

The above visual examination has merit in that it visually and intuitively shows how the nose pattern of a dog is formed and remains invariant, and shows that the nose patterns of different dogs differ. However, if one wants to compare all possible pairs in Figure A1, the number of comparisons needed is 4753, which would be a very laborious task. Moreover, the visual examination, however appealing, is a qualitative method. Therefore, to be more precise, one has to resort to a more quantitative method of biometric technology. Biometric algorithms and technology are, by now, very well developed. Its scope, aims and methodologies are well established and widely used. For more details, one may consult Introduction to Biometrics by Jain et al. [31], Encyclopedia of Biometrics by Li and Jain [32], or Animal Biometrics by Kumar et al. [33]. These books go into detail on how to take ROIs and how to create templates and perform matching, etc. The biometric method we use in this paper utilizes human iris recognition biometric algorithms, as in [34,35].

**Figure 2:**
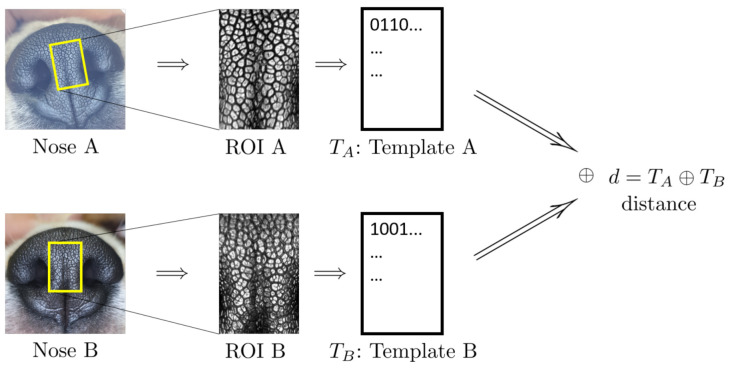
Schematics of Template Creation and Matching (Copyright 2021 by the authors).

Figure 2 shows the schematic outline of nose biometric template creation and matching. The leftmost column shows the nose images of the two dogs in question. The top one, Nose A, is the ninth mensiversary image of dog ID 01 and the bottom one, Nose B, is the tenth mensiversary image of dog ID 02.

From the ROI, a biometric template, or, in short, a template, is created by applying the well-known Gabor transform. A template is a rectangular array of 0s and 1s whose value at the location $\left(x_{0},y_{0}\right)$ is determined by the sign of the value of Gabor transform at the location $\left(x_{0},y_{0}\right)$. For more details, see [34,35].

The second column shows the ROI of each dog from which the template is created. Template $T_{A}$ is created from ROI A, and Template $T_{B}$ from ROI B. These two templates are then compared by using the well-known Hamming distance [34,35]. The resulting Hamming distance is called the matching distance, and is denoted by $d\left(A,B\right)=T_{A}⊕T_{B}$. This matching distance is usually called the matching distance between the two nose images Nose A and Nose B, although technically the matching distance computation involves only template $T_{A}$ of ROI A and $T_{B}$ of ROI B. This matching distance measures how similar these two templates, and hence the two nose images, are. In general, the smaller the matching distance is, the more similar the two nose images are; the greater the matching distance, the more dissimilar the two nose images.

Recall that the two noses, Nose A and Nose B, in Figure 2 belong to two different dogs (IDs 01 and 02). tTe matching distance d(A,B) of these two nose images is 0.5165. Next, if one replaces the bottom nose image with that of the tenth mensiversary image of dog ID 01 (which we call Nose C for convenience), while keeping the top image as Nose A, then the resulting matching distance d(A,C) between Nose A and Nose C is 0.1334. In view of the fact that Nose A and C belong to the same dog (ID 01), while Nose A and Nose B belong to different dogs (IDs 01 and 02), d(A,C) being smaller than d(A,B) is very natural.

Now, let us compute the matching distances between all pairs of nose images in Figure A1. As shown in Table 2, the total number of comparisons in this case is 4753, of which the number of *genuine comparisons* (matching nose images of the same dog) is 432, while the number of *impostor comparisons* (matching nose images of the different dogs) is 4321. The statistics of the entire matching distances are collected in Table 3.

As shown in Table 3, the maximum of the genuine matching distances is 0.3577, while the minimum of the impostor matching distances is 0.4254. This means that the matching distance between two nose images of the same dog is always smaller than that of any matching distance between two nose images of different dogs.

For convenience, let us take a number, say 0.4, and call it a threshold value, or, in short, a threshold. To paraphrase, any genuine matching distance is less than this threshold and any impostor matching distance is greater than this threshold.

This fact has a very important implication for the use of nose images (the nose pattern) as a biometric marker. Suppose one is presented with two nose images without knowing whether these images come from the same dog or from different dogs. Then, the comparison process outlined in Figure 2 can be run to compute the matching distance. If this matching distance is less than the threshold, i.e., 0.4, one can declare that these two images come from the same dog; if, on the other hand, the matching distance is greater than the threshold, i.e., 0.4, one then can declare that these two images come from different dogs. This decision process produces no error, because the matching distance of any genuine pair has to be less than the threshold and the matching distance of any impostor pair has to be greater than the threshold.

This genuine/impostor dichotomy can be paraphrased as:

- (Genuine comparisons) the nose pattern of a dog formed at the second mensiversary remains the same throughout the dog’s life;
- (Impostor comparisons) the nose patterns of different dogs are always different regardless of when the images are taken.

In other words, we have algorithmically verified the objectives spelled out at the beginning of Section 3 and, hence, that the nose pattern is an accurate and reliable biometric marker.

**Figure 3:**
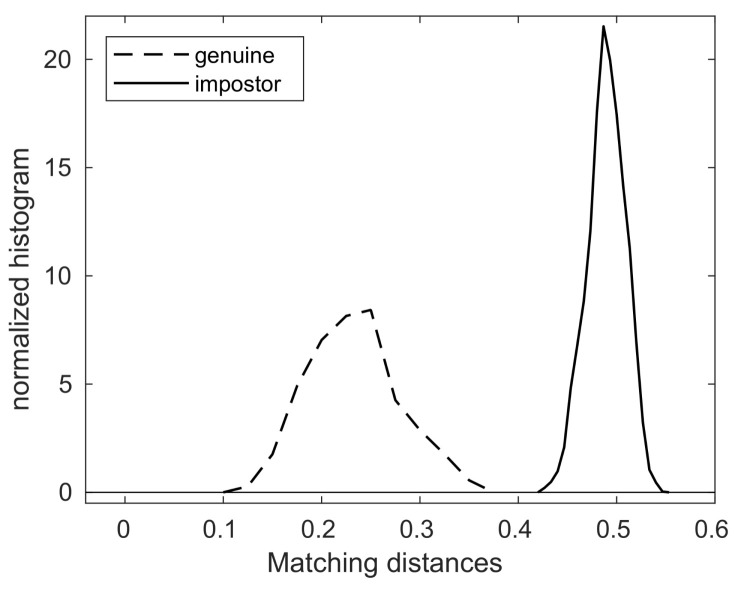
The probability distributions of genuine/impostor matching distances.

Figure 3 also shows the graphs of the normalized histograms (probability distributions) of genuine and impostor matching distances, and note that the threshold value 0.4 separates these two graphs of probability distributions.

## 5. Conclusions

In this paper, the nose images of ten beagle dogs were collected for the ten month period starting from the second mensiversary and ending at the eleventh mensiversary. Then the nose patterns of these images are examined visually and by a biometric algorithm. It is found that the canine nose patterns of these beagle dogs are fully formed at the second mensiversary and remains invariant until the eleventh mensiversary.

It should be noted that these puppies are siblings (six from one dam, the other four from another dam). Even so, our finding is that the nose patterns of genetically related dogs are clearly distinct, which is again a very strong evidence that the nose pattern is indeed unique to each individual dog. This result confirms and enhances the claims of earlier works by others [17,18,19,20] that the canine nose pattern is indeed unique to each animal. As a conclusion, our findings and those of others together imply that (1) the canine nose pattern is fully formed at the second mensiversary; and (2) this nose pattern stays invariant throughout a dog’s life to be used as a biometric marker.

Due to time limitations, we could not perform a longer-duration study. Therefore, the conclusion that the canine nose pattern remains invariant throughout a dog’s life is based on the understanding that biological traits remain stable from the early adult life until death. However, this is still a postulate which should be verified by a longer-duration study in the future.

The time period of this study starts at the second mensiversary for the specific purposes described in this paper. However, this is still a very interesting research topic to determine to the minute details, such as the earliest time at which the nose pattern is fully formed. We speculate that this timepoint is around the first mensiversary.

Finally, the primary concern of this paper is to study the intrinsic biological nature of nose pattern formation and invariance. When it comes to applying it for actual use in everyday life as a biometrical tool, there are many issues that have to be addressed separately. The most important is how to handle not-so-good images that occur in practice due to problems with camera focusing and the rapid movement tendencies of dogs. It is also important to make a nose-image-capturing device as user-friendly as possible. We hope to be able to return to these practical issues in our subsequent papers.

## Figures and Tables

**Table 1:** Dates of nose image capture.

Age in months	2 M	3 M	4 M	5 M	6 M
date	16 January 2020	20 February 2020	19 March 2020	23 April 2020	21 May 2020
Age in months	7 M	8 M	9 M	10 M	11 M
date	18 June 2020	16 July 2020	18 August 2020	15 September 2020	21 October 2020

**Table 2:** Comparison (matching) summary.

# of Subjects	10
# of Images per subject	9∼10
Total # of Images	98
# of Genuine comparisons	432
# of Impostor comparisons	4321

**Table 3:** Matching distance statistics.

Comparison Type	Min	Max	Mean	Std
genuine	0.1265	0.3577	0.2324	0.0456
impostor	0.4254	0.5496	0.4894	0.01955

## Data Availability

All data used in the paper are displayed as images in Figure A1 in Appendix A. Higher resolution images may be available for the purpose of academic study. Contact the corresponding author, Song-Hwa Kwon, at skwon@catholic.ac.kr for more details.

## Appendix A. Data

All image data used in this study are displayed in this Appendix section. Figure A1 shows all nose images and Figure A2, Figure A3, Figure A4, Figure A5, Figure A6, Figure A7, Figure A8, Figure A9, Figure A10 and Figure A11 are the corresponding ROI images. These ROI images are the original ROI images rotated and rescaled to make visual comparisons more intuitive and transparent.
